# Supplementary material for: Structural basis of the activation of TRPV5 channels by long-chain acyl-Coenzyme-A
Source: Nat Commun. 2023 Sep 21;14:5883. doi: 10.1038/s41467-023-41577-z (PMC10514044; doi:10.1038/s41467-023-41577-z)
Supplement: Supplementary file 1 — Supplementary Information [file 41467_2023_41577_MOESM1_ESM.pdf]

SUPPLEMENTAL INFORMATION

Structural basis of the activation of TRPV5 channels by long-chain acyl-Coenzyme-A

Bo-Hyun Lee, Jose J. De Jesus Perez, Vera Moiseenkova-Bell, Tibor Rohacs

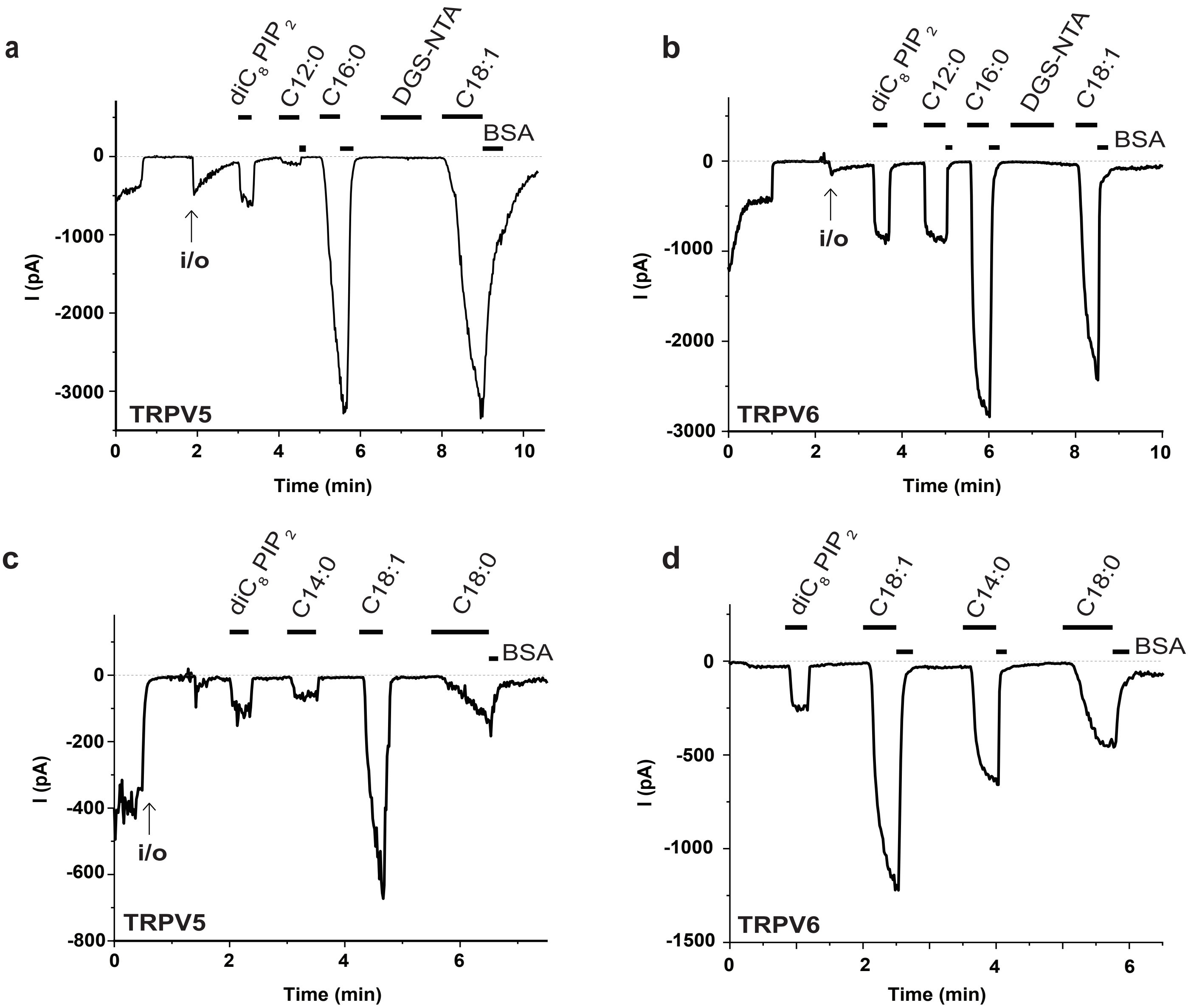

Supplementary Fig. 1. The effects of different LC-CoA species on TRPV5 and TRPV6 channels. Representative traces for Figure 1g,h. Excised inside-out patch clamp recordings from Xenopus oocytes expressing TRPV5 (a, c) and TRPV6 (b, d) were performed as described in the Methods. Traces show currents at -100 mV. Dashed lines show zero current. The establishment of the inside-out configuration is indicated by the arrows. The applications of 25 μM diC$_8$ PI(4,5)P$_2$ and 10 μM of different types of acyl-CoAs are shown by the horizontal lines.

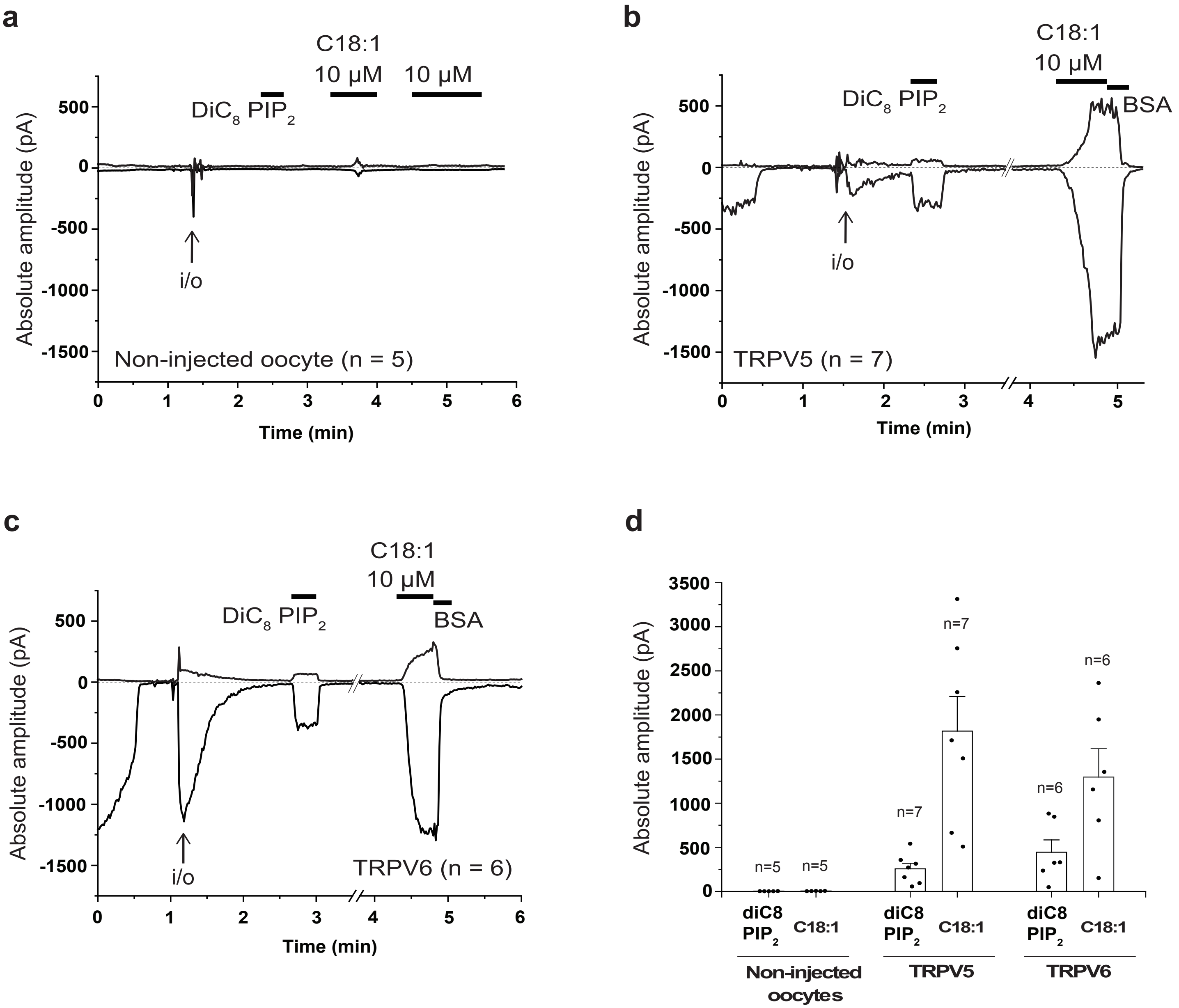

Supplementary Fig. 2. Oleoyl CoA does not induce currents in non-injected oocytes.
Excised inside-out patch clamp recordings in non-injected Xenopus laevis oocytes (a) and in TRPV5- and TRPV6-expressing oocytes (b,c) were performed as described in the Methods. Traces show currents at +100 mV (upper) and -100 mV (lower). Dashed lines show zero current. After excision, the patches were exposed to an air bubble to break any vesicle, such as in panel b and c, to establish the inside-out configuration (i/o). The applications of 25 µM diC$_8$ PI(4,5)P$_2$ and 10 µM oleoyl (C:18:1) CoA are indicated by the horizontal lines. d: data summary, mean ± S.E.M and scatter plots from the number of oocytes indicated in the figure from 2 independent oocyte isolations / injections.

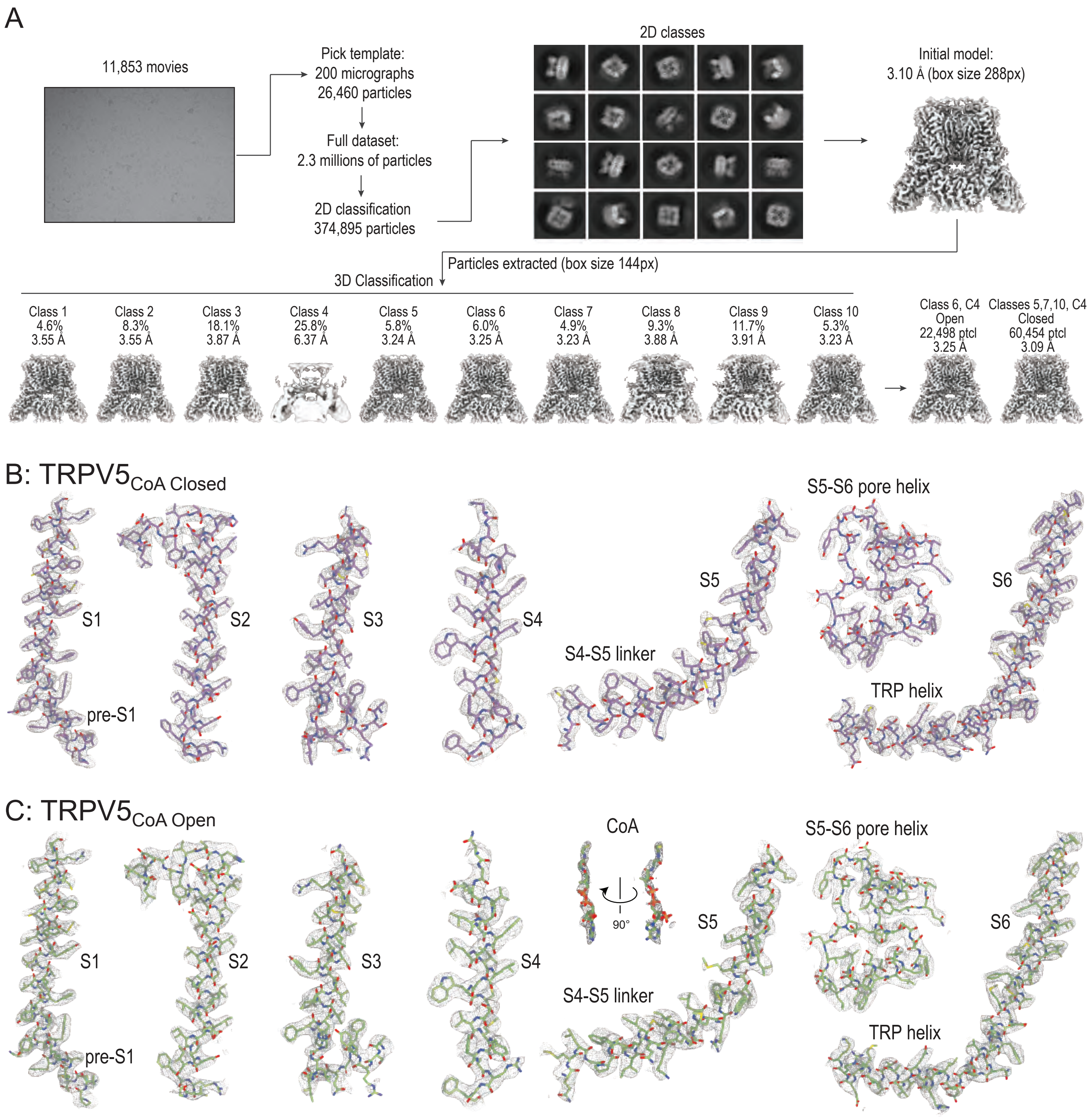

Supplementary Fig. 3. TRPV5$_{CoA\ Closed}$ and TRPV5$_{CoA\ Open}$ cryoEM data.
A: Data processing. Representative micrograph and 2D classes, initial model using a full box size (288px). 3D classes in C4 symmetry. B,C: Transmembrane domain densities of TRPV5$_{CoA\ Closed}$ (B) and TRPV5$_{CoA\ Open}$ (C) contoured at σ = 3.0.

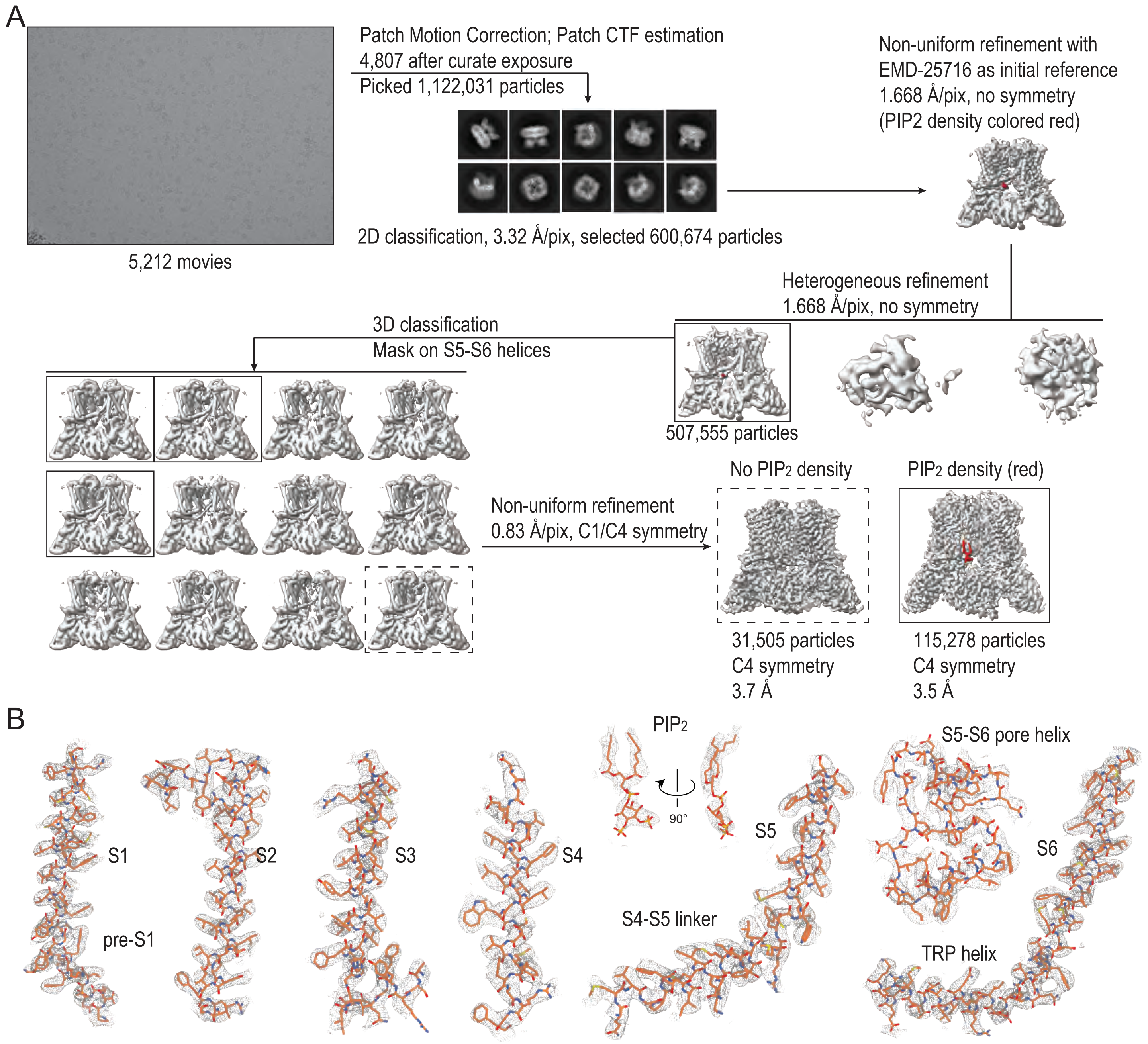

Supplementary Fig. 4. TRPV5$_{PIP2}$ cryoEM data.
A: Data processing. Representative micrograph and 2D classes, initial model, and 3D classes in C4 symmetry. B: Transmembrane domain densities of TRPV5$_{PIP2}$ contoured at σ = 3.0.

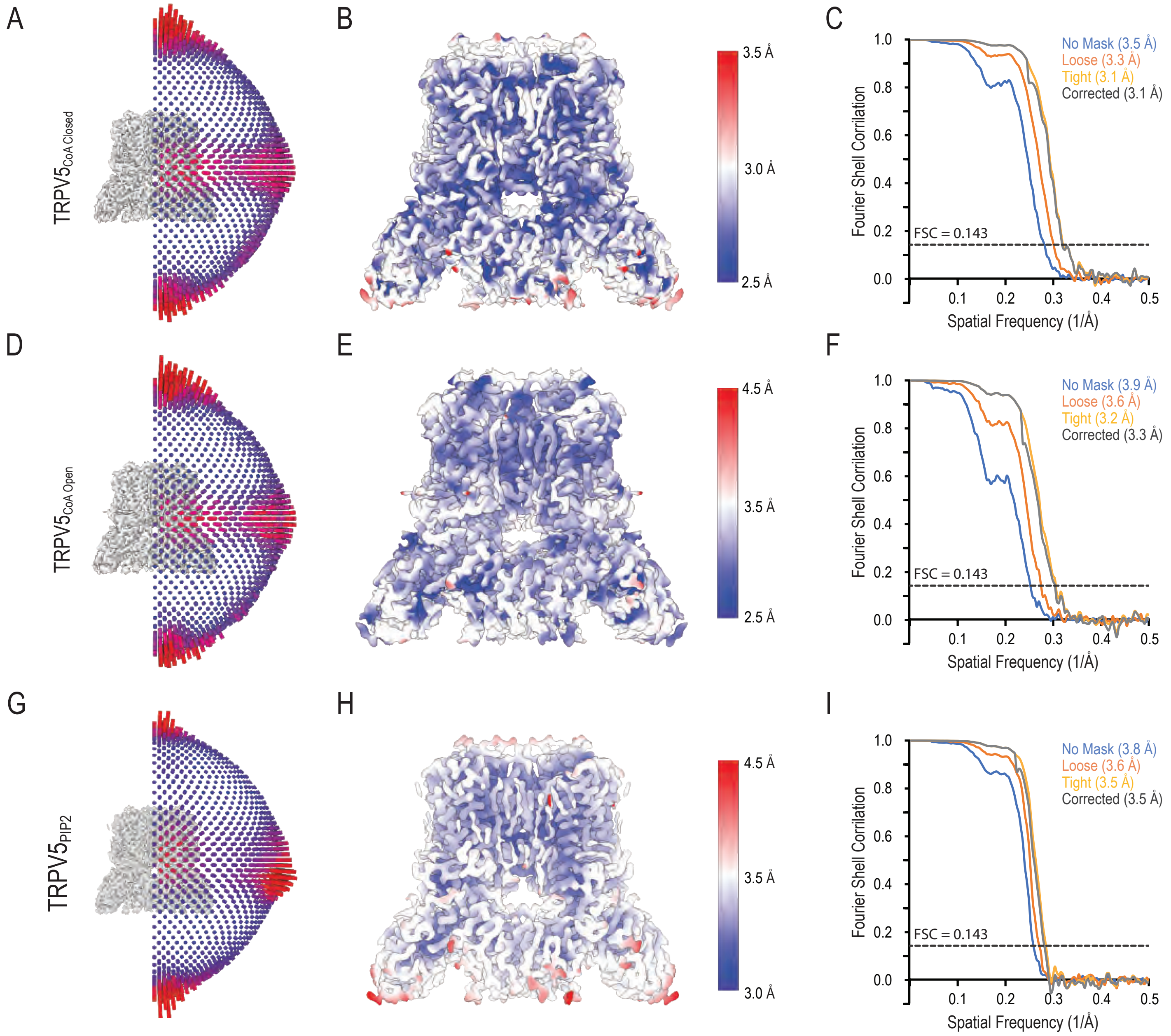

Supplementary Fig. 5. Angular distribution of final structures of TRPV5$_{\text{CoA Closed}}$ (A), TRPV5$_{\text{CoA Open}}$ (D), and TRPV5$_{\text{PIP2}}$ (G). Shorter blue cylinders represent less particles aligned at that angle; taller red cylinders indicate more particles. Local resolution and FSC curves estimated in cryoSPARC of TRPV5$_{\text{CoA Closed}}$ (B, C), TRPV5$_{\text{CoA Open}}$ (E, F), and TRPV5$_{\text{PIP2}}$ (H, I)
