# Supplementary material for: Structural basis of the activation of TRPV5 channels by long-chain acyl-Coenzyme-A
Source: Nat Commun. 2023 Sep 21;14:5883. doi: 10.1038/s41467-023-41577-z (PMC10514044; doi:10.1038/s41467-023-41577-z)
Supplement: Supplementary file 2 — Reporting Summary [file 41467_2023_41577_MOESM2_ESM.pdf]

nature portfolio

Corresponding author(s): Tibor Rohacs & Vera Moiseenkova-Bell

Last updated by author(s): Aug 25, 2023

Reporting Summary

Nature Portfolio wishes to improve the reproducibility of the work that we publish. This form provides structure for consistency and transparency in reporting. For further information on Nature Portfolio policies, see our Editorial Policies and the Editorial Policy Checklist.

Statistics

For all statistical analyses, confirm that the following items are present in the figure legend, table legend, main text, or Methods section.

n/a	Confirmed	
☐	☒	The exact sample size (*n*) for each experimental group/condition, given as a discrete number and unit of measurement
☐	☒	A statement on whether measurements were taken from distinct samples or whether the same sample was measured repeatedly
☐	☒	The statistical test(s) used AND whether they are one- or two-sided<br>*Only common tests should be described solely by name; describe more complex techniques in the Methods section.*
☒	☐	A description of all covariates tested
☐	☒	A description of any assumptions or corrections, such as tests of normality and adjustment for multiple comparisons
☐	☒	A full description of the statistical parameters including central tendency (e.g. means) or other basic estimates (e.g. regression coefficient) AND variation (e.g. standard deviation) or associated estimates of uncertainty (e.g. confidence intervals)
☐	☒	For null hypothesis testing, the test statistic (e.g. *F*, *t*, *r*) with confidence intervals, effect sizes, degrees of freedom and *P* value noted<br>*Give P values as exact values whenever suitable.*
☒	☐	For Bayesian analysis, information on the choice of priors and Markov chain Monte Carlo settings
☒	☐	For hierarchical and complex designs, identification of the appropriate level for tests and full reporting of outcomes
☒	☐	Estimates of effect sizes (e.g. Cohen's *d*, Pearson's *r*), indicating how they were calculated

Our web collection on statistics for biologists contains articles on many of the points above.

Software and code

Policy information about availability of computer code

Data collection	Clampex 11 (Molecular Devices) was used for collecting electrophysiology data.
Data analysis	Clampfit 11 (Molecular Devices), Origin 8 or Origin 2023 (OriginLab), were used to analyze electrophysiology data. The initial TRPV5 apo state model was adjusted manually in COOT. Real Space_Refinement, and eLBOW from the PHENIX software were used to refinement and to generate ligand restraints, respectively. Chimera, ChimeraX, and PyMol were used to analyze, visualize, and to generate figures. Pore profiles were determined using HOLE.

For manuscripts utilizing custom algorithms or software that are central to the research but not yet described in published literature, software must be made available to editors and reviewers. We strongly encourage code deposition in a community repository (e.g. GitHub). See the Nature Portfolio guidelines for submitting code & software for further information.

Data

Policy information about availability of data

All manuscripts must include a data availability statement. This statement should provide the following information, where applicable:

- Accession codes, unique identifiers, or web links for publicly available datasets
- A description of any restrictions on data availability
- For clinical datasets or third party data, please ensure that the statement adheres to our policy

We provided a data availablity statement. The atomic coordinates and cryo-EM density maps of the structures presented in this paper are deposited in the Protein

Data Bank and Electron Microscopy Data Bank under the accession codes TRPV5CoA Open (PDB 8FHI and EMD-29086), TRPV5CoA Closed (PDB 8FHH and EMD-29085), TRPV5PIP2 (8FFO, EMD-29049). Source data are provided with this paper.

Research involving human participants, their data, or biological material

Policy information about studies with human participants or human data. See also policy information about sex, gender (identity/presentation), and sexual orientation and race, ethnicity and racism.

Reporting on sex and gender	N/A
Reporting on race, ethnicity, or other socially relevant groupings	N/A
Population characteristics	N/A
Recruitment	N/A
Ethics oversight	N/A

Note that full information on the approval of the study protocol must also be provided in the manuscript.

Field-specific reporting

Please select the one below that is the best fit for your research. If you are not sure, read the appropriate sections before making your selection.

☒ Life sciences ☐ Behavioural & social sciences ☐ Ecological, evolutionary & environmental sciences

For a reference copy of the document with all sections, see nature.com/documents/nr-reporting-summary-flat.pdf

Life sciences study design

All studies must disclose on these points even when the disclosure is negative.

Sample size	Variance and effect size were not known before the study, thus no statistical method was used to predetermine sample sizes, but sample sizes were similar to those used on the field of electrophysiology. See for example Romero et al, Nature Communications 2023, PMID: 36859399
Data exclusions	Experiments with large leak currents and experiments where the seal broke before completion of the experiment were excluded,
Replication	Experiments generally were performed on several different oocyte preparations and several different transfections. The number of independent experiments are disclosed in the figures.
Randomization	Experiments generally were performed in a random order as much as the variable success rate of patch clamp experiments allow.
Blinding	Experiments were not blinded, as for electrophysiology measurements blinding is not practical, . See for example Neuberger et al Nature Communications 14(1):2451 2023: PMID:37117175. Also, analysis of electrophysiology data does not involve the level of subjectivity of behavioral experiments, where we routinely perform blinding.

Reporting for specific materials, systems and methods

We require information from authors about some types of materials, experimental systems and methods used in many studies. Here, indicate whether each material, system or method listed is relevant to your study. If you are not sure if a list item applies to your research, read the appropriate section before selecting a response.

Materials & experimental systems

n/a	Involved in the study	
☒	☐	Antibodies
☐	☒	Eukaryotic cell lines
☒	☐	Palaeontology and archaeology
☐	☒	Animals and other organisms
☒	☐	Clinical data
☒	☐	Dual use research of concern
☒	☐	Plants

Methods

n/a	Involved in the study	
☒	☐	ChIP-seq
☒	☐	Flow cytometry
☒	☐	MRI-based neuroimaging

Eukaryotic cell lines

Policy information about cell lines and Sex and Gender in Research

Cell line source(s)	HEK293 cells (CRL-1573) and HEK293T cells (#CRL-3216) were obtained from ATCC.
Authentication	HEK293 cells and HEK293T cells were authenticated by ATCC using STR profiling. We did not perform additional authentications.
Mycoplasma contamination	HEK293 cells and HEK293T cells were tested for mycoplasma, and were found negative.
Commonly misidentified lines (See ICLAC register)	No commonly misidentified cell line was used in this study.

Animals and other research organisms

Policy information about studies involving animals; ARRIVE guidelines recommended for reporting animal research, and Sex and Gender in Research

Laboratory animals	We used female Xenopus laevis frog to extract oocytes, to use as an expression system to study TRPV5 and TRPV6 channel activity.
Wild animals	No wild animals were used
Reporting on sex	The experiments were performed on isolated oocytes, thus sex is not a relevant variable.
Field-collected samples	We did not use filed-collected samples
Ethics oversight	Animal procedures were performed by protocols approved by the Institutional Animal Care and Use Committee at Rutgers New Jersey Medical School.

Note that full information on the approval of the study protocol must also be provided in the manuscript.
